# Clinical Experience of Poly‐L‐Lactic Acid (Sculptra) Across Different Patient Profiles Using the AART Methodology and Expert Consensus on Minimizing the Risk of Complications—The Malaysian Perspective

**DOI:** 10.1111/jocd.71165

**Published:** 2026-09-09

**Authors:** Stephanie Lam, Terry Lee, Doris Lee, Douglas Yuen, Sylvia Wai, Steve Chia, Thomas Low, Wei Thian Poh

**Affiliations:** ^1^ Central Health Medical Practice Central Hong Kong; ^2^ Klinik Terry Lee Kuala Lumpur Malaysia; ^3^ Sheen Clinic Subang Jaya Selangor Malaysia; ^4^ MX Clinic Johor Bahru Johor Malaysia; ^5^ Wai Clinic Puchong Selangor Malaysia; ^6^ Sliq Clinic Kuala Lumpur Malaysia; ^7^ MyClinic Petaling Jaya Selangor Malaysia; ^8^ Galderma Petaling Jaya Malaysia

**Keywords:** expert consensus, facial assessment scale, granuloma, nodules, poly‐L‐lactic acid, regenerative collagen biostimulator, vascular occlusion

## Abstract

**Background:**

Poly‐L‐lactic acid (PLLA‐SCA) is a well‐established regenerative collagen stimulator used for correction of shallow to deep nasolabial fold contour deficiencies, fine lines and wrinkles in the cheek region, and other facial wrinkles. Despite its widespread clinical use, variability in injection techniques and limited standardization contribute to inconsistent outcomes and potentially preventable complications. This paper presents expert recommendations for minimizing complications associated with biostimulators, including nodules, granulomas, and vascular occlusion. In addition, clinical cases demonstrating the application of the AART methodology with PLLA‐SCA treatment are presented based on author clinical experience.

**Methods:**

An expert consensus process was conducted involving six experienced aesthetic physicians specializing in biostimulator treatments. Consensus discussions focused on the identification, prevention, and management of complications associated with PLLA‐SCA, including nodules, granulomas, and vascular compromise. Four clinical cases demonstrating the utilization of AART methodology in routine practice were included.

**Results:**

The expert panel achieved consensus on key preventive strategies to minimize the risk of nodules, granulomas, and vascular occlusion, highlighting the importance of comprehensive anatomical assessment, precise injection techniques, appropriate product preparation, and proper post‐treatment care. Across all clinical cases, PLLA‐SCA treatment demonstrated improvements in Facial Assessment Scale (FAS) scores, Global Aesthetic Improvement Scale (GAIS) outcomes, and skin pinch test, with patient‐reported satisfaction with the treatment outcomes. No significant adverse events were observed in the presented cases.

**Conclusion:**

This expert consensus provides practical guidance for reducing complication risks associated with biostimulator injections in clinical practice. A thorough understanding of facial anatomy and adherence to appropriate injection protocols are essential to ensure optimal safety and efficacy. In addition, a structured approach to patient assessment and treatment planning using the AART methodology may assist practitioners in achieving consistent outcomes with PLLA‐SCA.

## 1 Introduction

The growing demand for regenerative aesthetic treatments has accelerated interest in biostimulatory agents capable of restoring skin quality through endogenous tissue remodeling rather than providing temporary volumisation. Sculptra (poly‐L‐lactic acid; PLLA‐SCA) is recognized as the first proven regenerative biostimulator, offering a mechanism of action that initiates deep, biologically driven skin renewal [1]. PLLA‐SCA is derived from plant‐based ingredients. Due to its unique manufacturing process, PLLA‐SCA exhibits a distinctive particle shape and size distribution with specific product characteristics. This manufacturing process is defined by nine key stages: growing, fermentation, polymerization, micronisation, particle size selection, low‐heat non‐residual sterilization, formulation with excipients, aseptic vial filling and freeze drying with hermetic sealing. The manufacturing process also includes quality assurance and control of all batches of PLLA‐SCA to ensure consistent quality and product characteristics. The PLLA‐SCA dry powder composition contains 150 mg poly‐L‐lactic acid with unique PLLA particles in shape and a mean particle size of 52 μm [2].

PLLA‐SCA has been extensively evaluated in multiple clinical trials, demonstrating efficacy lasting 18–24 months or longer [3]. Once injected, PLLA‐SCA activates a cascade of gene‐expression changes associated with anti‐inflammatory and reparative pathways, demonstrating distinct molecular advantages compared with CaHA‐R–based products and enabling early initiation of regenerative processes [4, 5]. In addition to stimulating neocollagenesis, studies have demonstrated that PLLA‐SCA is associated with activation of regenerative pathways as it stimulated more components of the extracellular matrix with less inflammatory response [5]. Molecular investigations reveal that PLLA‐SCA exerts epigenetic effects, including modulation of gene expression linked to elastin production, antioxidant pathways (MT1A), and adipocyte‐related metabolic activity (MARCO and VS1GA), thereby stimulating regenerative mechanisms across the dermal, hypodermal, and adipose tissue compartments [5, 6]. By stimulating Type I collagen production, regulating macrophage responses, and remodeling the extracellular matrix, PLLA‐SCA represents an advanced approach for personalized skin regeneration [5]. It delivers durable clinical outcomes that extend beyond superficial correction, with long‐lasting improvements consistently demonstrated in real‐world aesthetic practice [4, 7].

PLLA‐SCA demonstrates a well‐established safety and efficacy profile in clinical practice. As with all injectable biostimulatory agents, adverse events may occur, although their overall incidence remains low. Reported complications include delayed‐onset nodules, granulomatous reactions, and, rarely, vascular compromise. Nodule formation has been described in association with PLLA‐SCA; however, its occurrence is uncommon and has declined with the adoption of optimized reconstitution protocols, appropriate dilution volumes [8, 9], and refined injection techniques. Although the exact mechanisms underlying nodule formation remain unclear, several potential contributing factors have been proposed. These include particle size and its physicochemical properties, injection technique, product placement, inadequate post‐treatment massage leading to localized product entrapment, inexperienced injectors, and treatment in highly mobile facial areas [10, 11, 12]. Whilst PLLA‐SCA is considered both effective and generally safe, good technique and clinical judgment remain critical to minimize the risk of complications such as nodules, granulomas, and vascular occlusion.

In this context, this paper aims to present expert recommendations for minimizing the risk of complications, including nodules, granulomas, and vascular occlusion. It also describes the application of the AART (Assessment, Anatomy, Range, and Treatment) methodology in clinical practice for PLLA‐SCA treatment, based on four case studies. The AART methodology is a tool developed by Galderma for practitioners to perform a standardized full‐face assessment and to support an individualized treatment approach that holistically addresses a patient's aesthetic concerns [13]. Although the consensus addresses biostimulators in general, most of the panel's discussions focused on PLLA‐SCA, given its widespread clinical use and the availability of supporting evidence. These recommendations reflect the panel's opinions, grounded by their clinical experience with injectable procedures. The clinical insights discussed are based on Malaysia's clinical practice.

## 2 Methodlogy

### 2.1 Consensus Panel

Six qualified aesthetic physicians from private facilities across Malaysia, each with 8–10 years of clinical experience in biostimulator treatments, were invited and agreed to form an advisory panel.

### 2.2 Consensus Development

The consensus process was conducted based on the principles of the modified Delphi methodology, incorporating both a survey followed by a face‐to‐face meeting [14]. The survey comprising seven advisory statements was developed based on published literature. A non‐systematic literature search was conducted to support the development of the consensus statements. The literature was used to provide contextual background on the identification, diagnosis, and management of nodules and granulomas, as well as preventive strategies for complications such as vascular occlusion, including risk zone identification, safe injection planes, and injection techniques. All identified literature was used to guide the drafting of advisory statements and to support expert discussion during the consensus development process. A detailed consensus statement is provided in Appendix A.

#### 2.2.1 Round 1: Survey

The survey was distributed to panel members via the Google Forms platform (Google LLC, Mountain View, CA). The panel was asked to rate each statement using a five‐point Likert scale, from 1 (strongly disagree) to 5 (strongly agree), with the option to provide additional comments. Data collection was conducted anonymously, with no identifying questions included. Responses were subsequently collected and reviewed to identify areas of consensus and common viewpoints. Individual responses were not disclosed to other panel members, and only aggregated summary data were present and discussed during face‐to‐face meeting.

#### 2.2.2 Round 2: Face‐To‐Face Meeting

**FIGURE 1:**
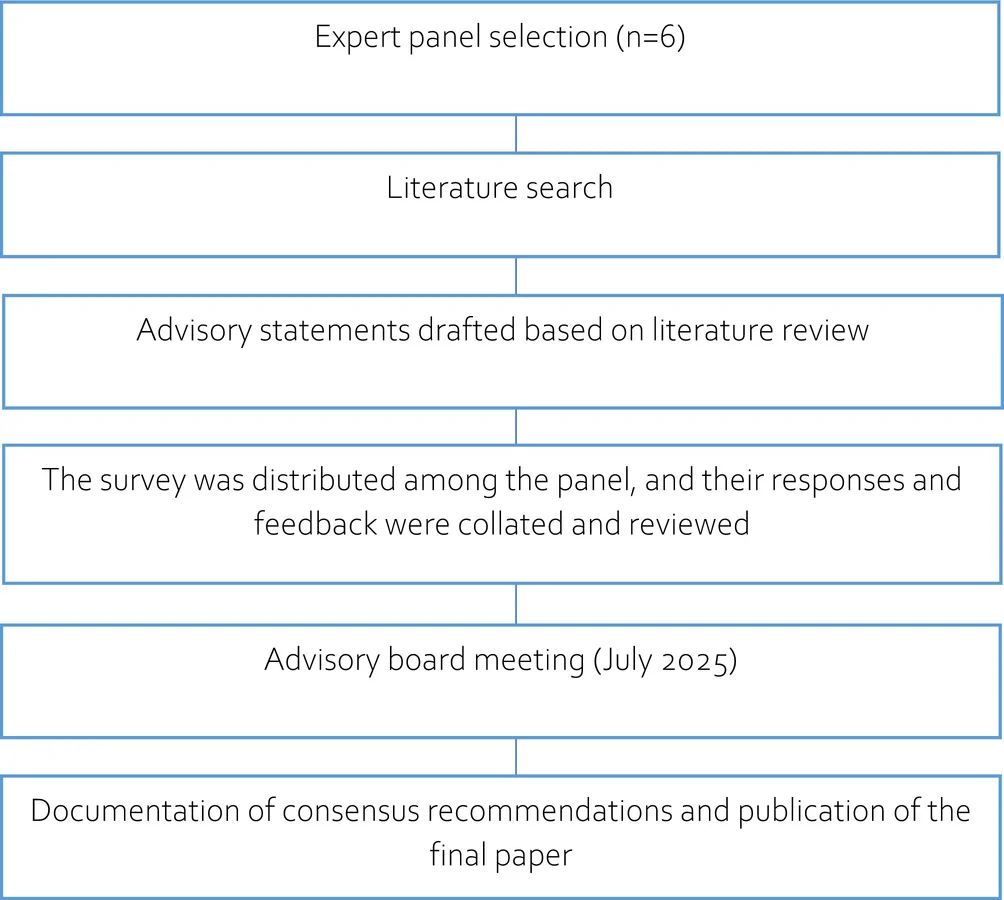
Process of developing consensus recommendations on minimizing risks of biostimulators.

In July 2025, all panel members participated in a face‐to‐face consensus meeting moderated by a chairperson who was a plastic surgeon with more than 10 years of experience in biostimulator treatments. During the meeting, aggregated results and panel feedback from the previously distributed survey were presented and discussed to facilitate clarification, interpretation, and refinement of the statements. However, statements that did not achieve consensus in Round 1 were not subjected to formal re‐voting. Instead, these items were discussed to improve clarity and to ensure a shared understanding among panel members. The final recommendations reflected the collective expert interpretation derived from both the survey results and the consensus discussion. Additionally, new statements formulated during the meeting were voted on, and the outcomes were incorporated into this paper. The consensus process is illustrated in Figure 1.

#### 2.2.3 Consensus Definition

Consensus was defined as agreement among all six experts (100%), strong agreement as agreement among five of the six experts (83.3%), and majority agreement as agreement among at least four of the six experts (66.7%). Agreement was defined as responses of “Agree” or “Strongly Agree” corresponding to Likert scores of 4 and 5.

### 2.3 Case Studies

**FIGURE 2:**
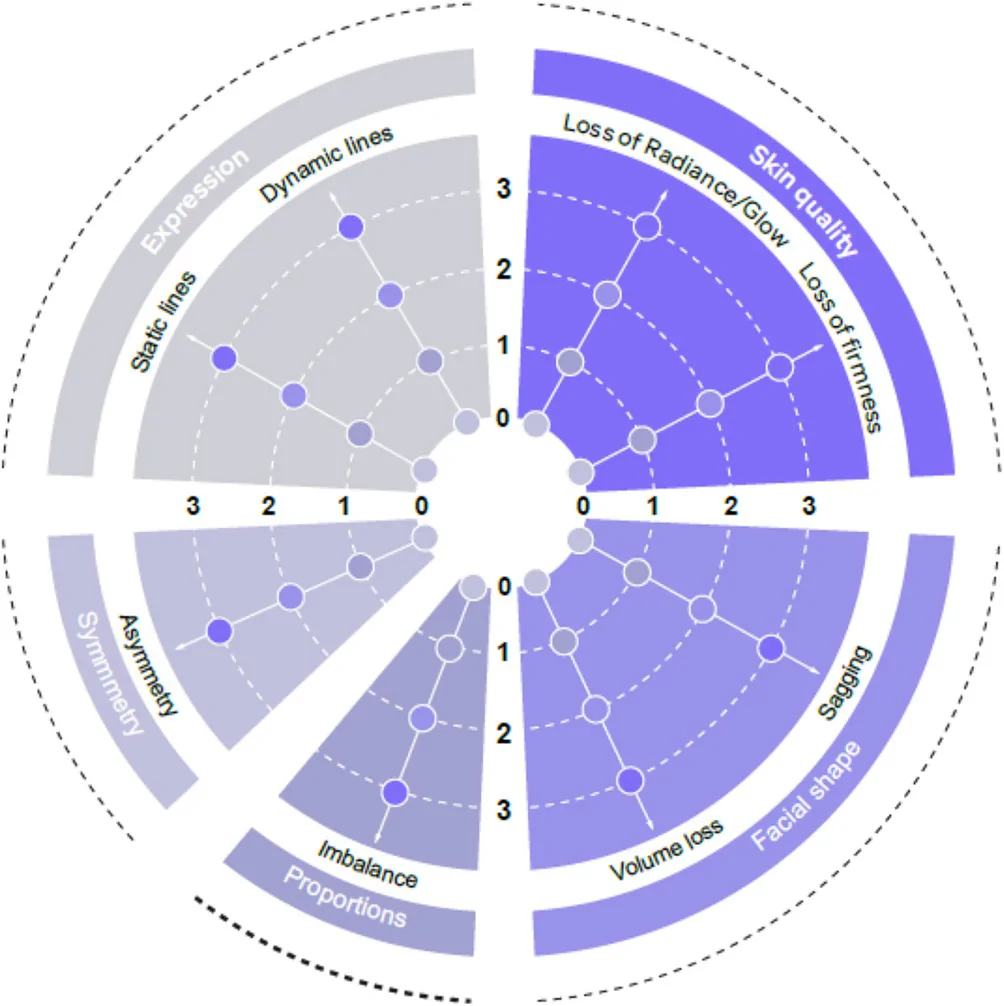
The Facial Assessment Scale (FAS).

A series of case studies was presented to describe the application of the AART methodology in assisting facial assessment and treatment planning for PLLA‐SCA. The AART method consists of facial assessment conducted using the Facial Aesthetic Score (FAS) (Figure 2). The five domains assessed included skin quality, facial shape, facial proportion and contour, facial symmetry, and animation and emotional expression. Each domain was graded using a four‐point scale (0 = none, 1 = mild, 2 = moderate, 3 = severe).

Treatment outcomes were evaluated based on changes in FAS scores, the Global Aesthetic Improvement Scale (GAIS), and subjective assessment using the pinch test to evaluate skin firmness. GAIS is a five‐point global improvement scale, where 1 indicates “worsened,” 2 “no change,” 3 “improved” (mild change), 4 “much improved” (noticeable improvement), and 5 “very much improved” (marked improvement). The skin pinch test was assessed based on whether an improvement in skin firmness was observed. In addition, patient satisfaction was recorded at the end of treatment, while adverse events were monitored throughout the treatment period. Prior to treatment, patients were informed of the potential risks and benefits and provided written informed consent for the publication of their clinical data and photographs.

## 3 Results

### 3.1 Identification, Diagnosis and Management of Biostimulator Complication

**FIGURE 3:**
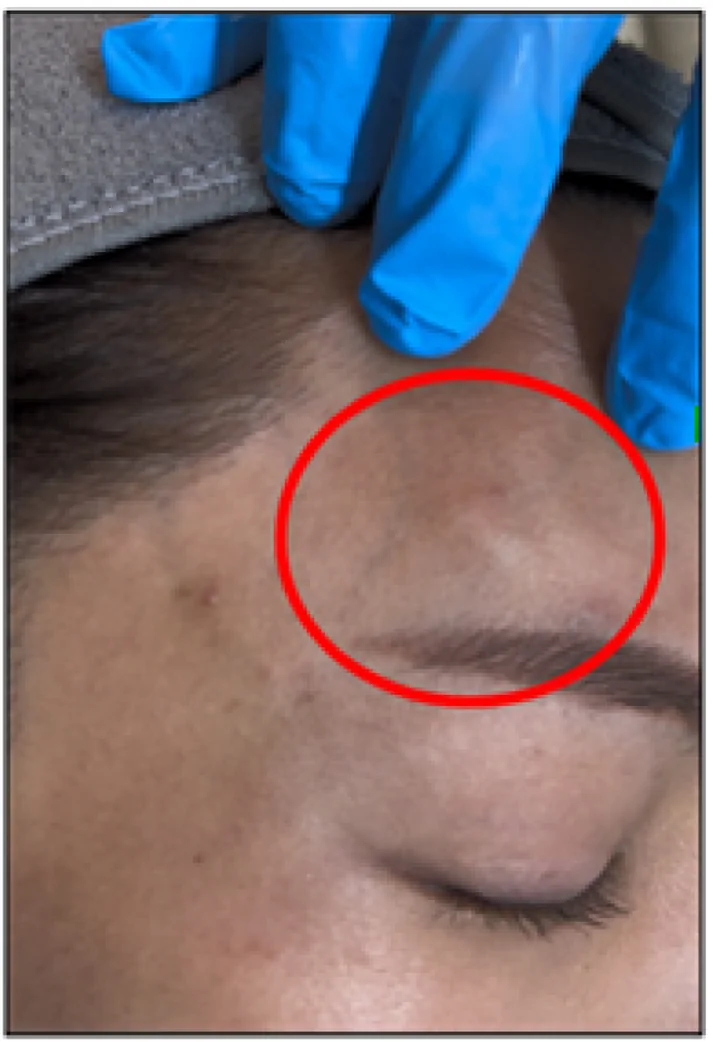
Clinical photograph showing non‐inflammatory nodules on the right side of the forehead prior to treatment. Image courtesy of Dr. Doris Lee. **Management of nodules using mechanical dispersion:** the case describes a 45‐year‐old woman who developed two non‐inflammatory, palpable nodules on the forehead 1 week after receiving PLLA‐SCA injections in the frontalis region. The nodules were treated with two sessions of normal saline mixed with lignocaine (9:1 ratio), with approximately 5 mL administered into each nodule per session, followed by gentle massage. The treatments were performed 2 weeks apart. The patient was advised to continue massage at home for at least 2 weeks. Complete resolution of the nodules was achieved after the second session.

**FIGURE 4:**
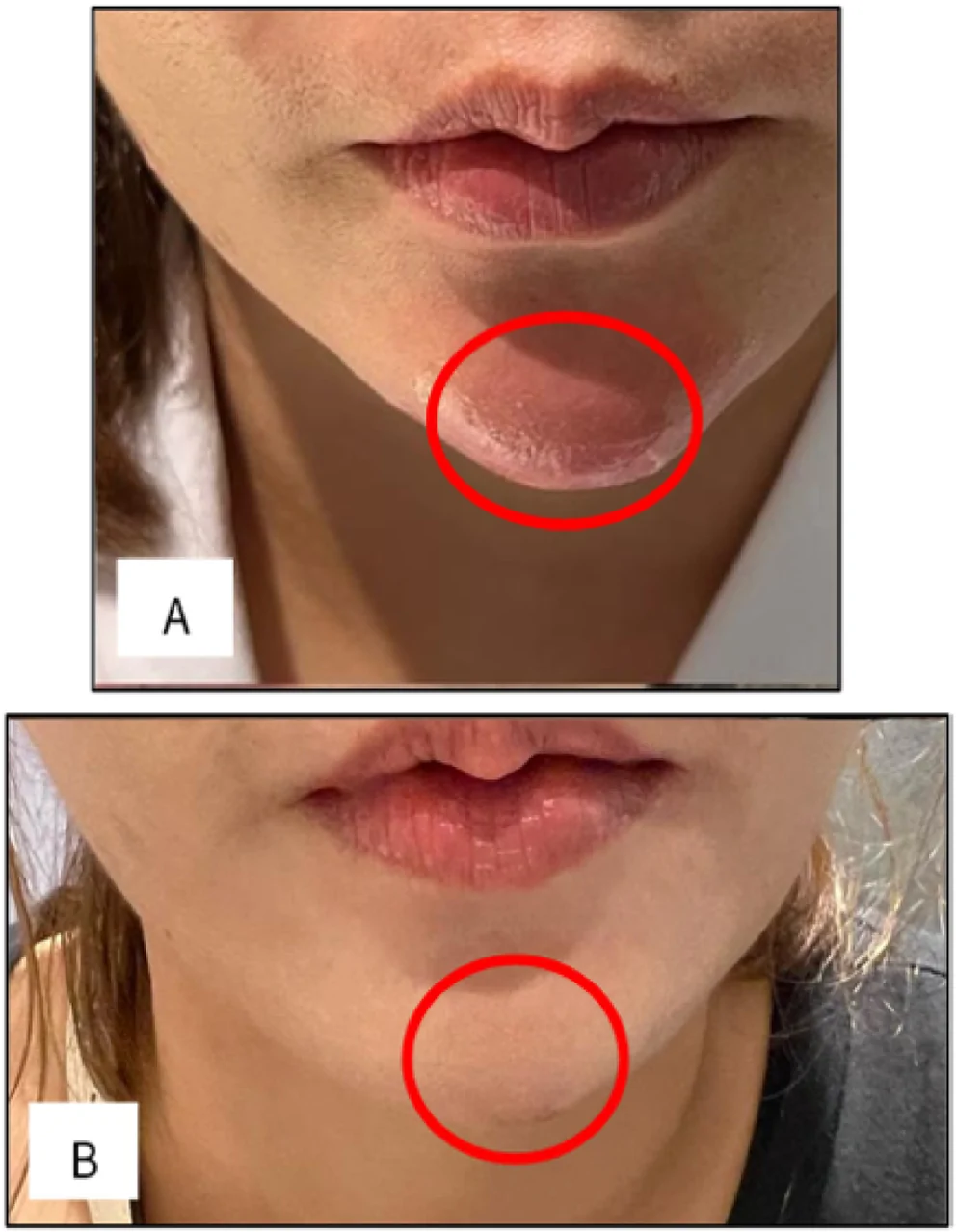
Clinical photographs of a patient presenting with a persistent lump on the chin (A). The nodule was no longer visible or palpable 2 months later (B). Images courtesy of Dr. Terry Lee. **Management of nodules/granulomas using energy‐based devices:** the case involved a 41‐year‐old woman who presented with a persistent, palpable inflammatory nodule on the chin for 2 years following an unknown biostimulator injection, suspected to result from an excessive inflammatory or immune‐mediated response. Surgical excision had failed, with no histopathology available and culture results were negative. Subsequent treatment was performed with Endolift, a laser‐assisted technique, using the LASEmaR 1500 device, which operates at 1470–1500 nm. The nodule was reduced by approximately 50% after the first session and was no longer visible or palpable after the second session, 2 months later. No atrophy of surrounding tissue was observed, unlike what is sometimes seen with steroid treatment. However, the effectiveness of energy‐based devices may vary depending on the biostimulator type.

**FIGURE 5:**
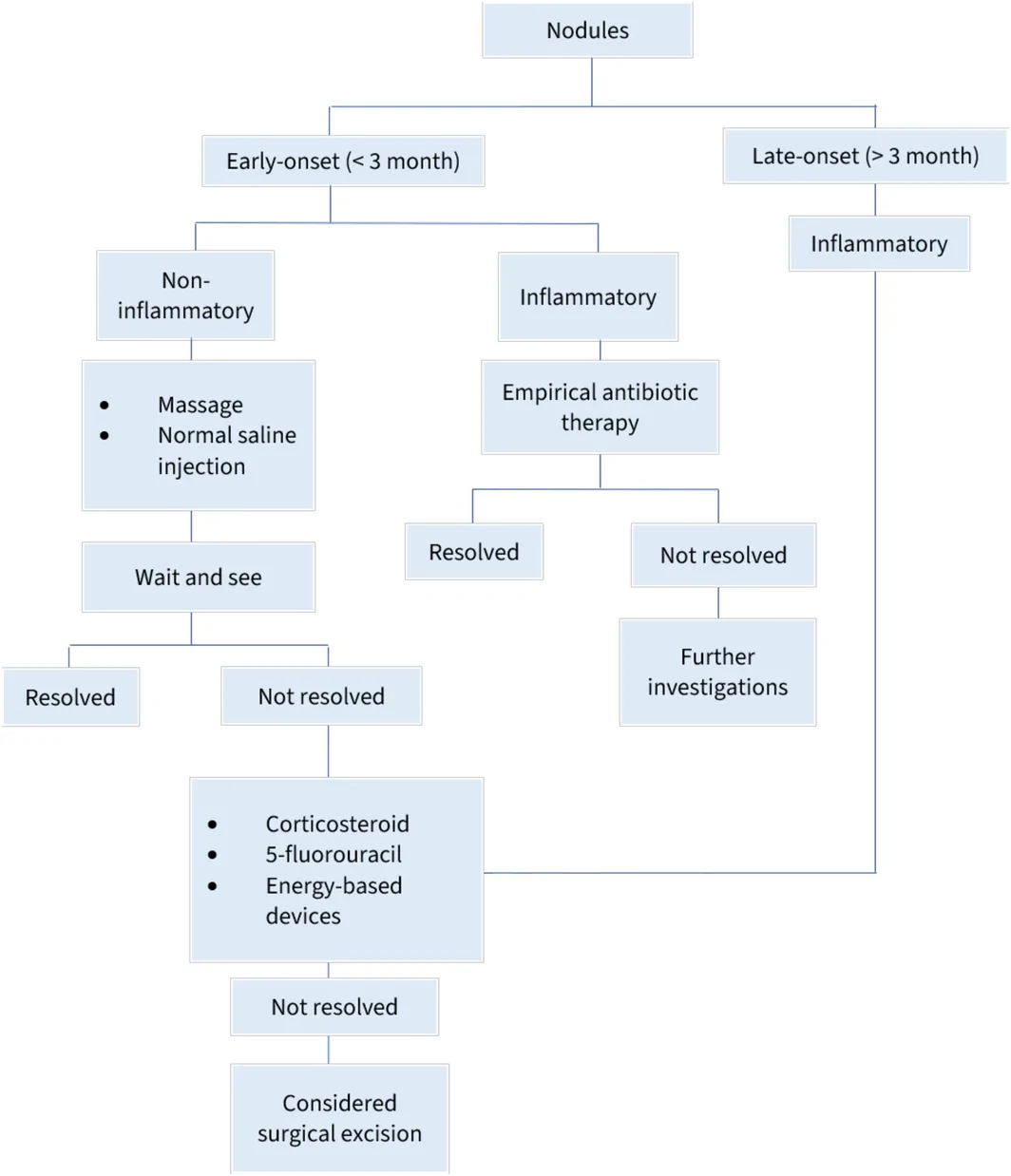
Algorithm for the management of nodules and granulomas following PLLA‐SCA treatment.

The panel reached agreement (83.4%) on recognizing the symptoms, with 100% consensus on diagnosis, management, and preventative measures to minimize nodules and granulomas associated with biostimulator use. While the recommendations were intended to apply broadly across available biostimulators, most of the panel's insights, particularly those related to injection techniques, dilution protocols, and complication management, were primarily derived from clinical experience with PLLA‐SCA. Table 1 presents the recommendations for which agreement was reached among the panelists. To demonstrate approaches in managing nodules and granulomas, clinical examples using mechanical dispersion and an energy‐based device are presented in Figures 3 and 4, respectively. However, the management case using an energy‐based device represents an isolated clinical observation derived from one of the author's clinical experiences. This finding is exploratory in nature and should be interpreted with caution, as it does not establish clinical efficacy or support standardized treatment recommendations. Rather, it provides preliminary clinical insight that warrants further investigation in controlled studies. In addition, Figure 5 presents a proposed management algorithm illustrating how nodules and granulomas can be addressed.

**TABLE 1:** Recommendations on the identification, diagnosis, management, and preventative measure of nodules and granulomas.

Recognizing the Symptoms of Nodules and Granulomas
Nodules may be visible or only palpable, are typically firm and localized, and can present with or without inflammatory signs, often resulting from technical errors or uneven product distribution.
Granulomas are identified as visible or palpable nodules that are usually accompanied by inflammatory features, typically resulting from an excessive inflammatory response.
Nodules generally occur within the first 3 months after treatment, whereas granulomas tend to develop later.
Diagnosis of Nodules and Granulomas
Diagnosis is based on the onset of nodules after treatment and whether they are inflammatory or non‐inflammatory. The type of biostimulator used should be identified.
Detailed clinical history is essential for an accurate diagnosis.
Bacteriological testing and histopathological confirmation of granulomas are generally not recommended, due to the rarity of infection following PLLA‐SCA treatment and the limited practicality of these investigations in clinical practice. While routine bacteriological testing and histopathological confirmation are not required for all nodules, these investigations should be considered in selected cases such as suspected infection, biofilm formation, atypical or progressive inflammatory features, treatment resistance, or diagnostic uncertainty.
Management of Nodules and Granulomas
For early‐onset inflammatory nodules, empirical antibiotic therapy is recommended as the first‐line treatment.
Early‐onset non‐inflammatory nodules are initially managed with mechanical dispersion using normal saline administered via needle or cannula, followed by clinical observation.
Ultrasound guidance may be used, where available, to assist in product dispersion.
Early‐onset nodules generally respond well to appropriate management.
In cases of late‐onset nodules or when nodules do not resolve with mechanical dispersion, intralesional corticosteroids, 5‐fluorouracil (5‐FU), or energy‐based devices may be employed, either alone or in combination.
Surgical intervention is considered only if the above treatment options are ineffective.
Preventative Measure of Nodules and Granuloma
Subcutaneous or subdermal planes are preferred for injections.
Supraperiosteal, intramuscular, intradermal, and interfascial plane injections should be avoided, particularly when using PLLA‐SCA.
Given the increased risk of nodule formation associated with injections in the central facial regions, particularly in areas of high dynamic movement, treatment should be restricted to the lateral facial regions.
The use of a cannula is recommended to help ensure that injections remain within the correct anatomical plane.
The product should be well‐diluted, homogeneous, and injected evenly over a wide area to ensure uniform distribution while avoiding bolus injections.

### 3.2 Minimizing the Risk of Vascular Occlusion

The incidence of vascular occlusion associated with biostimulators is considered very low, particularly with PLLA‐SCA. Young et al. reported two published cases of vascular occlusion following PLLA‐SCA treatment: one involving injection to the periorbital/nasal region and another to the temple [15, 16, 17]. All panel members agreed that, to date, no cases of blindness related to biostimulator use, particularly PLLA‐SCA, have been reported in Malaysia. Based on their clinical experience, the panel considered that the risk of vascular occlusion, including retinal artery occlusion, may be lower with PLLA‐SCA due to its water‐based suspension compared with gel‐based biostimulators. Despite the low incidence, the panel strongly recommended strict adherence to established safety practices. Consensus recommendations on preventative measures to minimize the risk of vascular occlusion are summarized in Table 2.

**TABLE 2:** Consensus recommendations on preventative measures for vascular occlusion.

Preventative Measure for Vascular Occlusion
Understand facial vascular anatomy and safe injection techniques.
High‐risk areas such as the glabella, nose, nasolabial folds, and forehead should be approached with caution. Treatment in these regions should be performed by a well‐trained healthcare professional with advanced anatomical knowledge and experience in managing potential complications.
Adhere to sterile technique, use a blunt cannula (22G–25G), inject slowly and evenly. Aspiration prior to injection may be performed as a precautionary measure; however, it is not a definitive method to prevent intravascular injection.
Monitor for early warning signs, such as blanching at the injection site or pain distant from the injection area.
Review the patient's medical and procedural history, as prior procedures may have altered arterial anatomy.

### 3.3 Identification of Risk Zones and Safe Injection Techniques

**FIGURE 6:**
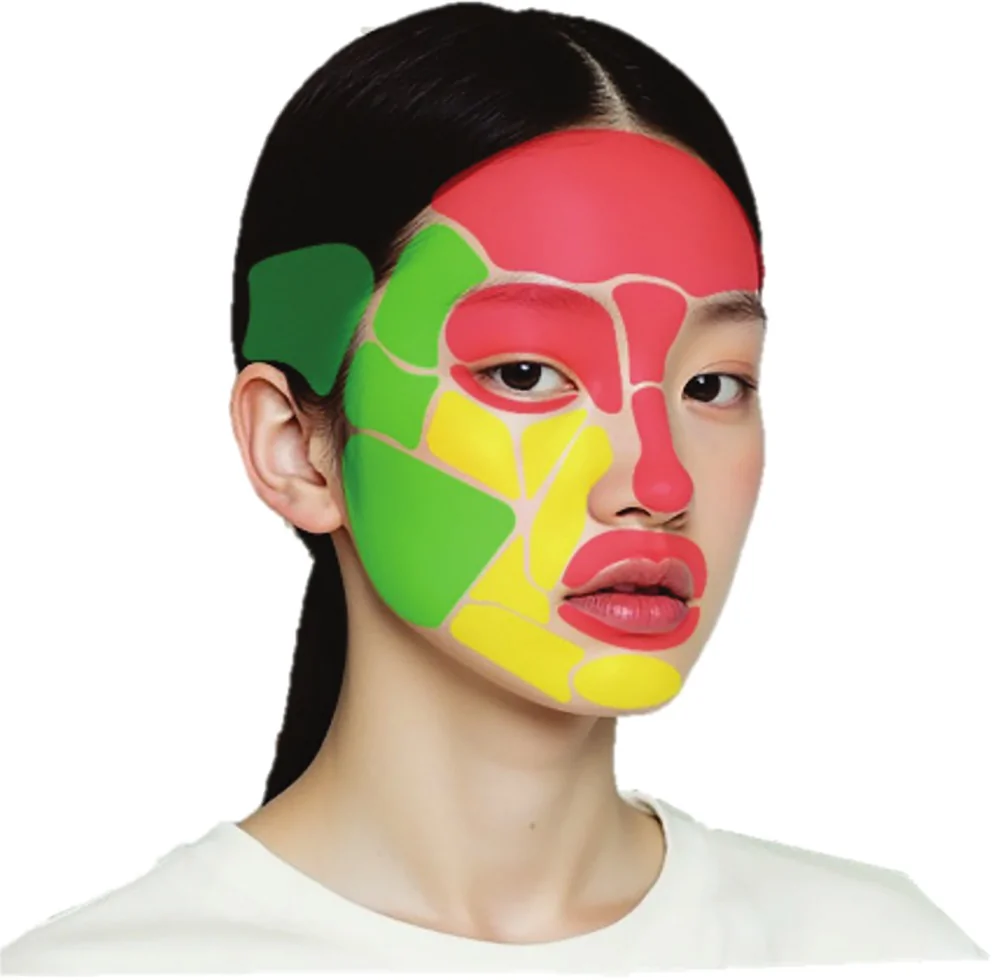
Facial areas categorized by the risk of vascular occlusion: High (red), intermediate (yellow), and low (green).

**FIGURE 7:**
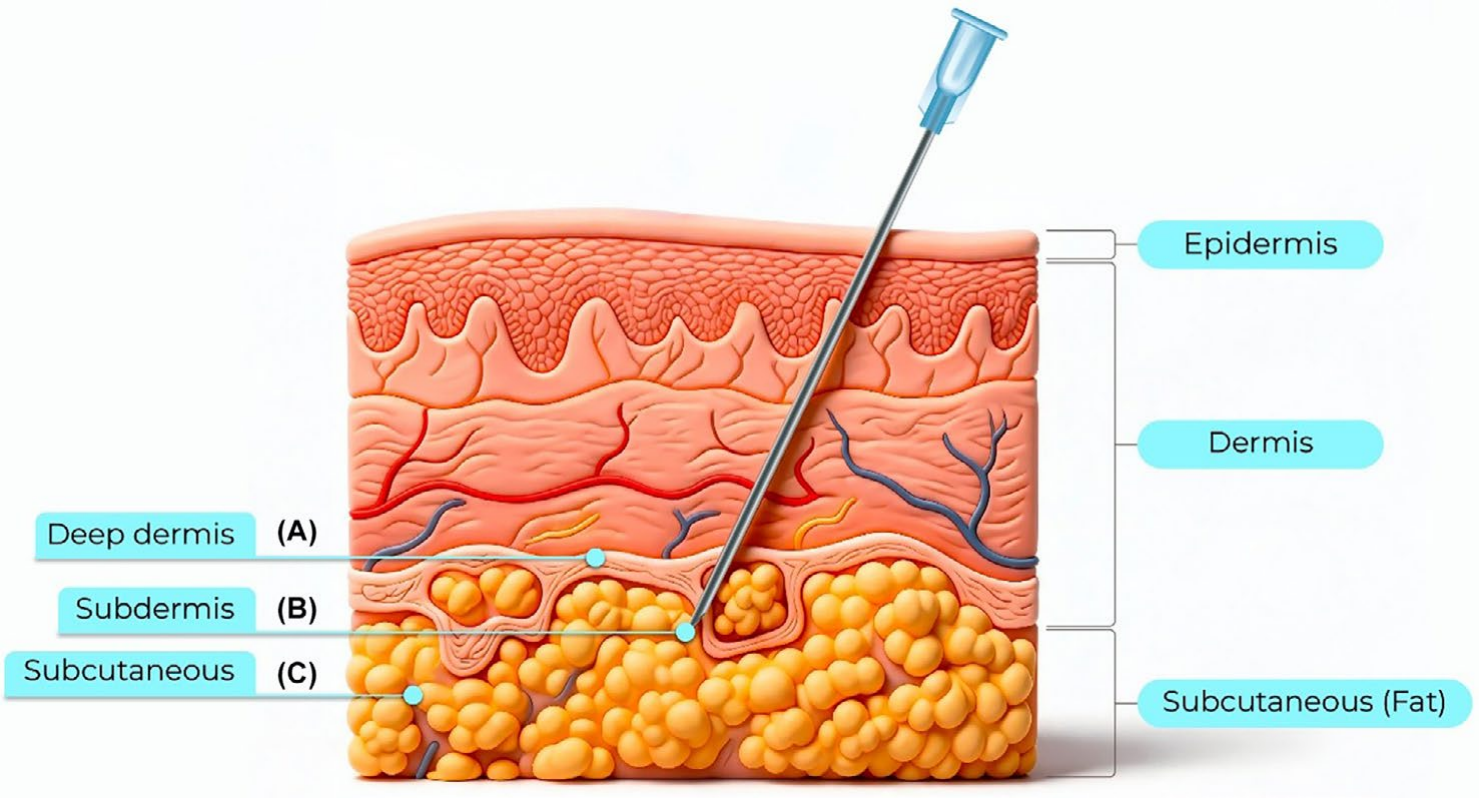
A schematic diagram presenting anatomical planes used for PLLA‐SCA injection. (A) Deep dermis: Injection placed in the deeper portion of the dermal layer, above the dermo‐subcutaneous junction; (B) Subdermal: Injection delivered immediately beneath the dermis at the dermal–subcutaneous junction; (C) Subcutaneous: Injection placed within the superficial or deep adipose tissue layer below the dermis.

To further assist practitioners in minimizing the risk of nodules, granulomas, and vascular occlusion associated with biostimulator use, the panel identified risk zones for vascular occlusion (Figure 6) and recommended injection planes and techniques to reduce these adverse events (Table 3). No consensus was reached regarding the risk level of the chin, with half of the panel classifying it as intermediate risk and the other half as high risk. In addition, a schematic diagram of different anatomical injection planes relevant to PLLA‐SCA is presented in Figure 7 to assist practitioners in understanding the various injection planes.

**TABLE 3:** Anatomical regions, risk levels, injection planes, and techniques for biostimulator.

Area		High risk	Intermediate risk	Low risk	Injection plane	Injection techniques
Frontal region	Forehead	✓			Not recommended	
Temporal region	Anterior Temple			✓	Deep dermis Subcutaneous	Retrograde linear threading
	Posterior Temple			✓	Deep dermis Subcutaneous	Retrograde linear threading
Periorbital region		✓			Not recommended	
Nasal region		✓			Not recommended	
Cheeks	Lateral cheek			✓	Deep dermis Subcutaneous	Retrograde linear threading
	Medial cheek		✓		Deep dermis Subcutaneous	Retrograde linear threading
	Middle cheek			✓	Deep dermis Subcutaneous	Retrograde linear threading
	Nasolabial folds		✓		Not recommended	
Lip and perioral region		✓			Not recommended	
Chin			✓		Not recommended	
Mandibular area	Inferior jowl/area along mandibular border		✓		Deep dermis Subcutaneous	Retrograde linear threading

### 3.4 Case Studies on the Application of the AART Methodology

Four patient cases were presented to demonstrate the application of the AART methodology in clinical practice. Table 4 summarizes the patient profiles and treatment protocols for each case.

**TABLE 4:** Summary of patient profiles and treatment protocols.

Variables	Patient 1	Patient 2	Patient 3	Patient 4
Age/Gender	52/Female	47/Female	38/Female	49/Female
PLLA‐SCA facial treatment profiles	Skin quality and youthful glow	Renewed volume	Filling lift	Slimming lift
Treatment areas	Bilateral: submalar region	Bilateral: Temporal region, lateral cheek	Bilateral: Temporal region, mid‐cheek	Bilateral: Posterior temple, temple, subzygoma, cheek, submalar regions
Injection plane	Subdermal	Subdermal	Subdermal & subcutaneous	Subdermal & subcutaneous
Cannula size	22G	25G	25G	23G
Dilution	8 mL SWFI +1 mL lidocaine	8 mL SWFI +1 mL lidocaine	8 mL SWFI +1 mL lidocaine	8 mL SWFI +1 mL lidocaine
Total vials/volume	3 vials/27 mL	4 vials/36 mL	5 vials/45 mL	6 vials/54 mL
Total volume	S1–S3: 9 mL	S1: 18 mL S2 and S3: 9 mL	S1 and S2: 18 mL S3: 9 mL	S1–S3: 18 mL
Injection distribution	S1–S3: 4.5 mL (submalar)	S1: 4.5 mL (temporal region & lateral cheek) S2 and S3: 2.0 mL (temporal region) + 2.5 mL (lateral cheek)	S1 and S2: 4 mL (temple) + 5 mL (mid‐cheek) S3: 4.5 mL (temples + mid‐cheek)	S1–S3: 4 mL (posterior temple) + 2 mL (temple) + 2 mL (subzygoma) +1 mL (cheek)
Number of sessions	3 sessions	3 sessions	3 sessions	3 sessions
Treatment session/interval	Baseline, Week 6, Month 4	30‐day intervals	2‐month intervals	8‐week intervals

Abbreviations: S, session; SWFI, sterile water for injection.

#### 3.4.1 Patient 1: Skin Quality and Youthful Glow

**FIGURE 8:**
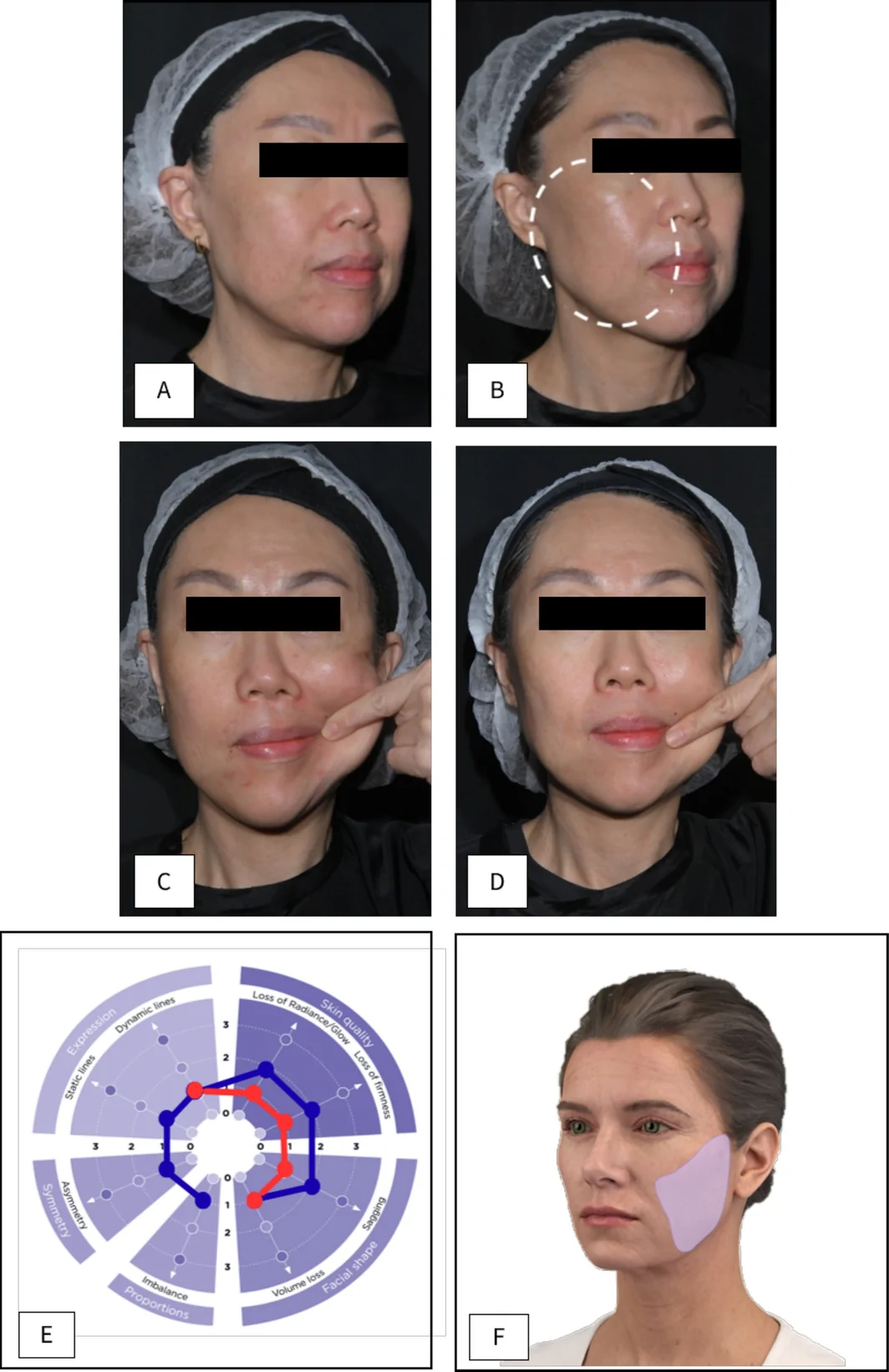
Clinical photographs demonstrating patient improvement from baseline (A) to 4 months (B) following treatment; Pinch test photograph at baseline (C) and at 4 months (D) post‐treatment; Corresponding FAS scores at baseline (blue line) and 4 months post‐treatment (red line) (E); Targeted injection areas for this profile: Cheeks, submalar, or temple regions (F). Images courtesy of Dr. Douglas Yuen.

The patient presented with concerns of dry skin, uneven skin texture, enlarged pores, and smile lines. Baseline FAS assessment demonstrated moderate scores for skin radiance, firmness, and facial sagging. Following treatment, improvement in skin quality was observed (Figure 8B), with GAIS scores of 4, indicating perceived improvement in aesthetic appearance. FAS scores for skin radiance, firmness, and facial sagging decreased from moderate to mild (Figure 8E), with improvement in the pinch test observed (Figure 8D). The patient reported satisfaction with the treatment outcomes, noting perceived improvement in skin glow and radiance. No adverse events were reported throughout the treatment period.

#### 3.4.2 Patient 2: Renewed Volume

**FIGURE 9:**
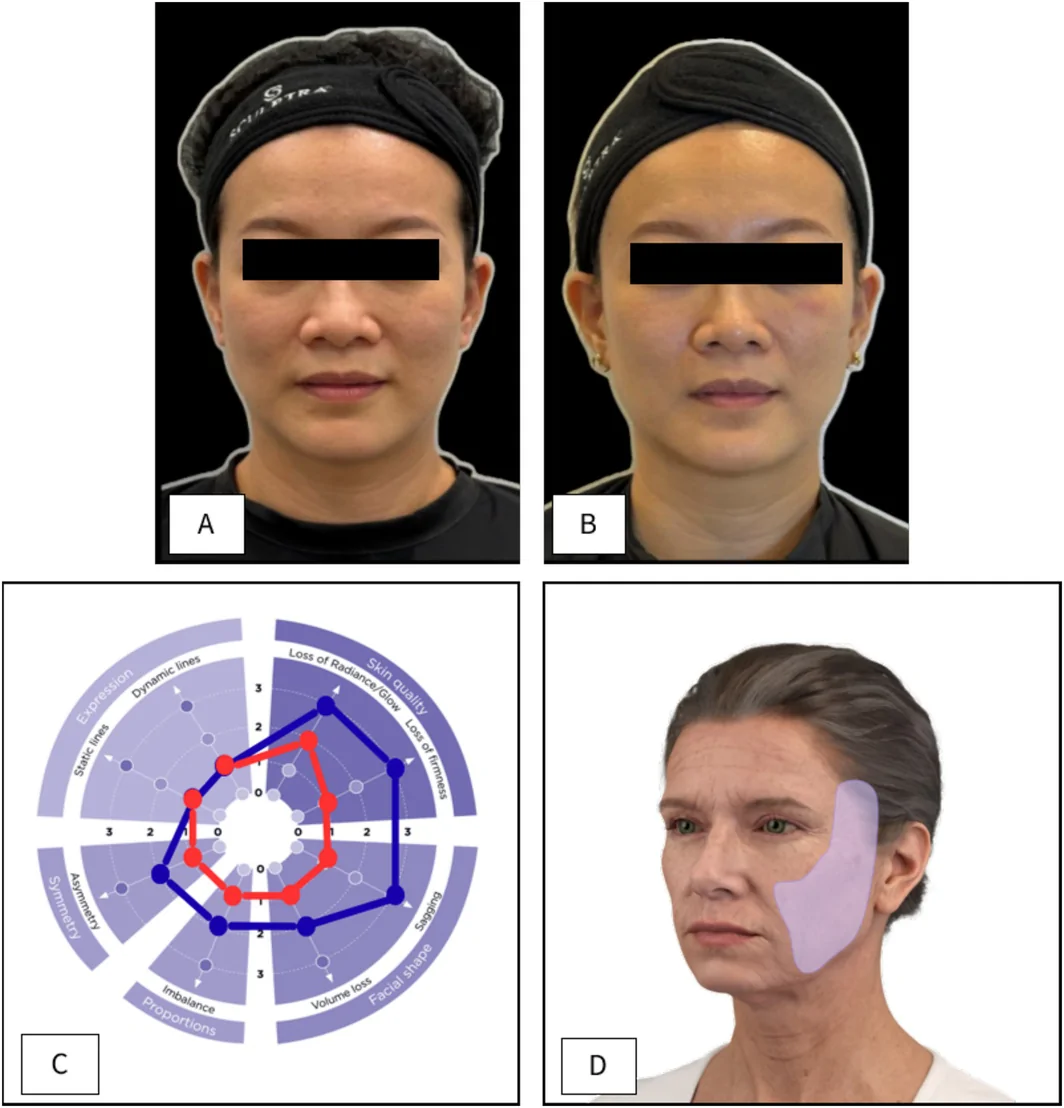
Clinical photographs demonstrating patient improvement from baseline (A) to 2 months (B) following treatment; Corresponding FAS scores at baseline (blue line) and 2 months post‐treatment (red line) (C); Targeted injection areas for this profile: Submalar and anterior temples (D). Images courtesy of Dr. Terry Lee.

The patient presented with a history of significant weight loss and loss of firmness and volume. FAS assessment demonstrated a severe score in the skin quality domain and facial sagging, with moderate scores for volume loss, proportion imbalance, and facial asymmetry. Following treatment, clinical observation demonstrated visible volume restoration, improved mid‐face projection and contour definition (Figure 9B), with GAIS scores of 4, indicating perceived improvement in aesthetic appearance. A reduction in FAS scores was observed across all affected domains (Figure 9C). The patient reported satisfaction with the treatment outcomes and perceived improvement in volume loss. No adverse events were reported throughout the treatment period.

#### 3.4.3 Patient 3: Filling Lift

**FIGURE 10:**
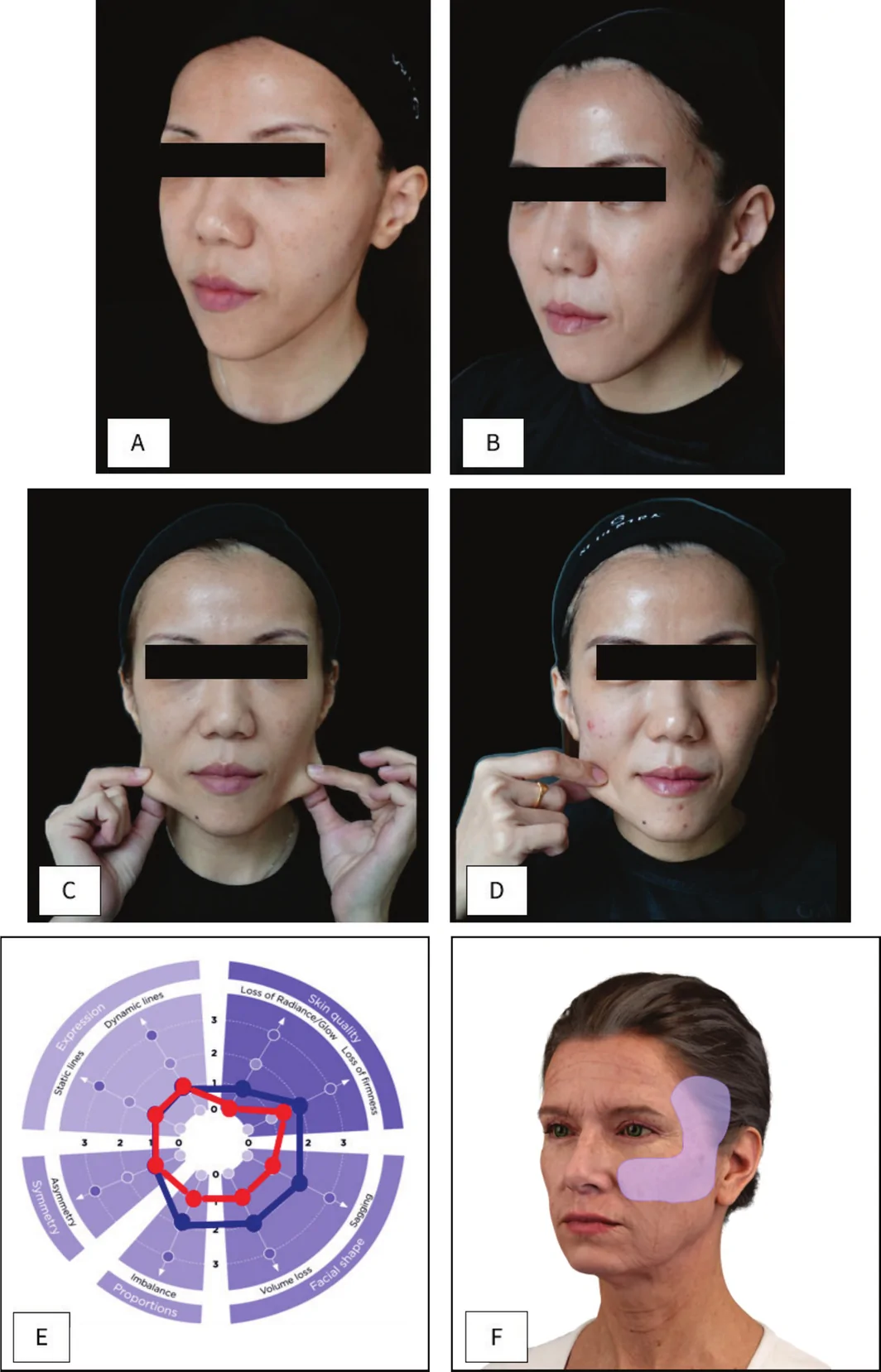
Clinical photographs demonstrating patient improvement from baseline (A) to 9 months (B) following treatment; Pinch test photograph at baseline (C) and at 3 months (D) post‐treatment; Corresponding FAS scores at baseline (blue line) and 9 months post‐treatment (red line) (E); Targeted injection areas for this profile: Zygomatic and temporal regions (F). Images courtesy of Dr. Steve Chia.

The patient presented with midface deflation characterized by zygomatic volume loss and sagging. FAS assessment revealed mild‐to‐moderate scores in the skin quality domain and moderate scores in the facial shape and proportion domains. Following treatment, visible lifting with restoration of midface volume was observed clinically (Figure 10B), with GAIS scores of 4, indicating perceived improvement in aesthetic appearance. Reduction in FAS scores was observed in the skin quality domain, facial shape and proportion domains (Figure 10E), and improvement was noted in the pinch test (Figure 10D). The patient reported satisfaction with the treatment outcomes. No adverse events were reported throughout the treatment period.

#### 3.4.4 Patient 4: Slimming Lift

**FIGURE 11:**
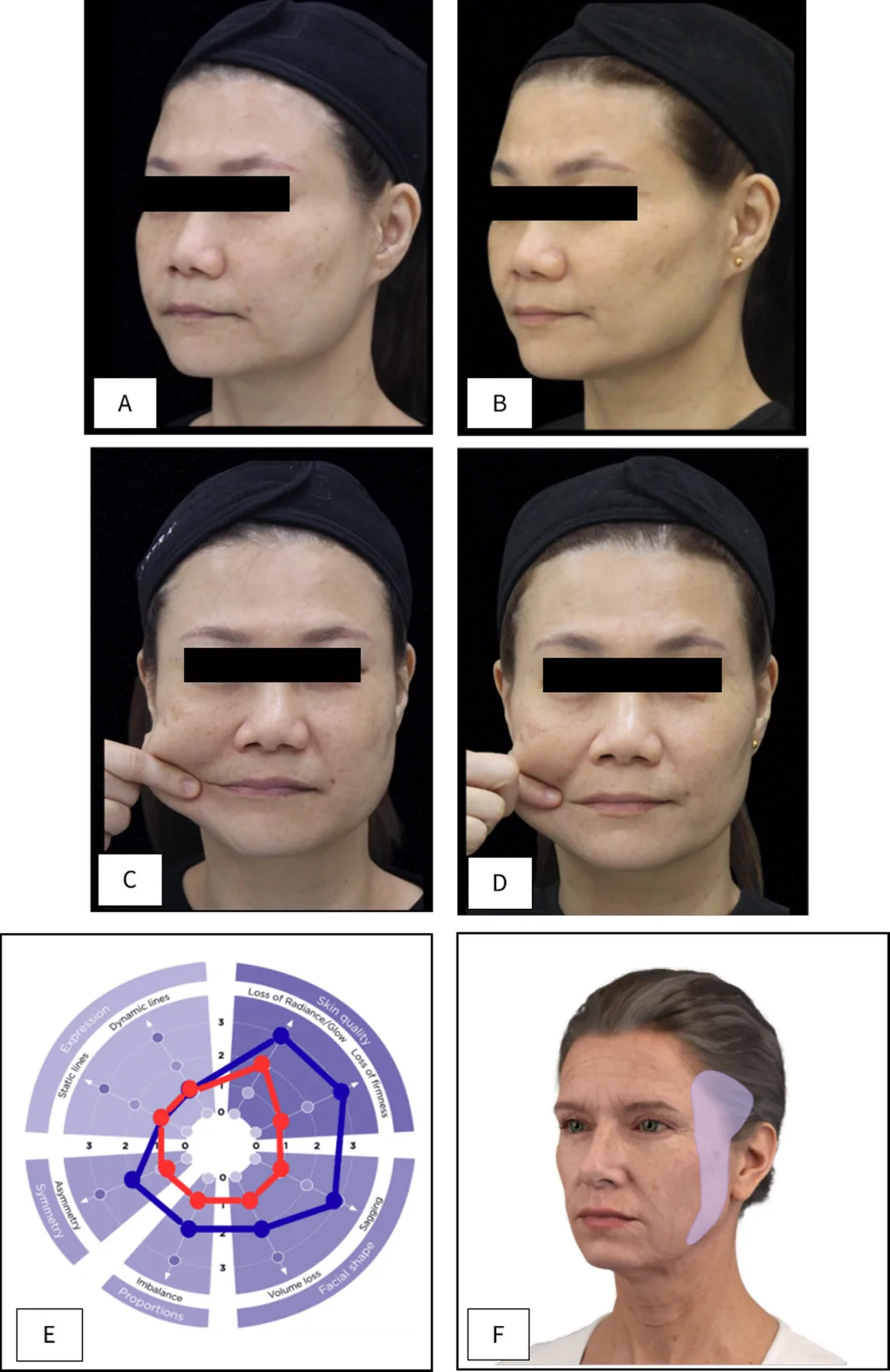
Clinical photographs demonstrating patient improvement from baseline (A) to 6 months (B) following treatment; Pinch test photograph at baseline (C) and at 6 months (D) post‐treatment; Corresponding FAS scores at baseline (blue line) and 6 months post‐treatment (red line) (E); Targeted injection areas for this profile: Pre‐auricular, anterior/posterior temporal region (F). Images courtesy of Dr. Doris Lee.

The patient presented with a heavy, rounded facial appearance with loss of firmness, facial sagging, disproportion, and asymmetry. FAS assessment revealed a severe score in the skin quality domain and severe‐to‐moderate scores in the facial shape, proportion, and facial asymmetry domains. Following treatment, improvement in facial proportion and jawline contouring was observed clinically (Figure 11B), with GAIS scores of 4, indicating perceived improvement in aesthetic appearance. Improvement in FAS scores from severe to moderate or mild was observed for all relevant domains (Figure 11E), with improvement in the pinch test reported (Figure 11D). The patient reported satisfaction with the treatment outcomes and perceived a noticeable improvement in facial appearance. No adverse events were reported throughout the treatment period.

## 4 Discussion

This paper provides expert consensus recommendations to minimize complications associated with PLLA‐SCA treatment, including nodules, granulomas, and vascular occlusion, while illustrating the practical application of the AART methodology across four representative patient profiles. The four patient cases demonstrate how the AART framework may be incorporated into routine clinical practice to support individualized treatment planning. Clinical improvements, including reductions in FAS scores and improvements in overall aesthetic appearance, were observed in these cases, with patients reporting satisfaction following treatment.

Although most biostimulators, particularly PLLA‐SCA, have a well‐established safety profile with a relatively low incidence of complications, practitioners must remain vigilant for potential adverse events and adopt appropriate preventive measures. The expert consensus presented in this paper provides practical, clinically relevant recommendations for minimizing the risk of nodules, granulomas, and vascular occlusion, reflecting real‐world practice in Malaysia.

Consistent with existing literature, the panel emphasized that the diagnosis and management of nodules and granulomas are determined by the timing of onset and the presence or absence of inflammatory features. Early, non‐inflammatory nodules are generally associated with uneven product distribution, superficial placement, or injection of excessive volumes [18, 19]. These nodules are often resolved with conservative management, including clinical observation and mechanical disruption [20, 21, 22, 23]. In contrast, late onset nodules or granulomas reflect a chronic inflammatory response to foreign material that cannot be effectively cleared by macrophages [18]. Management may include intralesional corticosteroids, 5‐fluorouracil, or energy‐based devices, alone or in combination with surgical excision, which is considered only if conservative measures fail [24, 25, 26, 27, 28, 29, 30].

Proper injection technique and thorough anatomical knowledge are essential to minimize the risk of nodules and granulomas. Superficial placement should be avoided in hypermobile areas, such as hypermimetic or concentric muscle regions, and in regions with multiple muscle insertions, including the oral commissures [31]. Similarly, deep injections, including supraperiosteal, intramuscular, intradermal, and interfascial planes, are not recommended with PLLA‐SCA, as they may lead to unintended product deposition and migration, increasing the likelihood of nodule formation.

Panel members highlighted that the incidence of nodules with PLLA‐SCA has been extremely low in recent years, a trend attributed to the adoption of new dilution protocols, including immediate use after reconstitution [20]. The panel therefore advocates reconstitution of PLLA‐SCA with 8 mL SWFI and 1 mL lidocaine, with immediate administration after reconstitution to minimize the risk of nodules. Additionally, total injection volume for the anterior and posterior temples should be limited to 4.5 mL per session. For patients with significant volume deficiency, smaller volumes administered over multiple sessions are preferred to higher volumes in a single session, ensuring both safety and optimal outcomes.

Although PLLA‐SCA is associated with a low incidence of vascular occlusion, adherence to established safety principles remains critical. The panel's recommendations in this paper align with a recent review by Young et al., which emphasizes the importance of understanding facial anatomy and proper injection techniques to minimize the risk of vascular occlusion from biostimulator injectables [15]. Injections should be performed slowly, preferably using a blunt cannula, with low volumes and the smallest effective amount of product, while carefully monitoring the patient's pain response. Retrograde injection with continuous needle or cannula motion is also recommended to avoid accumulation of product at a single location [15].

While aspiration prior to injection does not reliably prevent intravascular injection, it is still recommended as a precautionary measure [15, 32]. Obtaining a patient's medical and procedural history during the initial consultation, particularly regarding prior aesthetic procedures or surgeries, is also essential [15]. This is important because areas with previous surgical reconstruction or trauma may present an increased risk due to altered or newly formed anastomotic vascular pathways [16, 33]. Furthermore, practitioners must be able to recognize the early clinical signs of ischaemia and ensure that clear management protocols and referral pathways are established to facilitate timely intervention should complications arise [34].

### 4.1 Other Adverse Events and Recommendations

Mild to moderate soreness at the injection site is common on the first day following PLLA‐SCA administration. Compared with other biostimulators, PLLA‐SCA generally causes minimal swelling, as it induces a lower inflammatory response. No severe infections were reported by panel members following PLLA‐SCA treatment. However, a minor entry‐site infection was noted in one case, likely associated with improper aftercare. To minimize such risks, the following aftercare measures are recommended when performing massages after PLLA‐SCA treatment:

- Use appropriate dermocosmetic products during massage.
- Avoid applying facial oils, serums, or heavy cosmetics over the injection site for the first 3 days.
- Keep the area clean and avoid water contact for 2 to 3 h after the procedure.
- Maintain proper hygiene when performing the recommended “5‐5‐5” massage routine (5 min, five times per day for 5 days after the injection session).

PLLA‐SCA treatment should be postponed in patients with unexplained urticaria, active inflammation, or recent allergic reactions, as performing the procedure during an active inflammatory state may exacerbate the response to PLLA‐SCA. Caution is also advised in patients with a history of nodule formation from other biostimulators, as they may have an increased risk of developing nodules. Nevertheless, with adequate dilution, proper injection technique, and appropriate treatment timing, favorable outcomes can still be achieved.

## 5 Conclusion

This paper highlights expert panel recommendations, including practical strategies and preventive measures to minimize complications associated with biostimulator treatment and to assist practitioners in preventing and managing potential adverse events. Adherence to appropriate product preparation, injection techniques, and aftercare recommendations is expected to support safe clinical practice and optimize treatment outcomes. In addition, the case studies provide practical examples of how the AART methodology may be incorporated into routine clinical practice to support individualized treatment planning with PLLA‐SCA treatment.

## Author Contributions

All authors were involved in the development and finalization of the expert consensus. Terry Lee, Doris Lee, Douglas Yuen, and Steve Chia contributed clinical patient data and relevant clinical experience. All authors participated in reviewing the manuscript, providing critical intellectual feedback, and approving the final version for submission.

## Funding

This work was supported by the Galderma.

## Ethics Statement

This manuscript presents expert consensus and clinical experience based on routine clinical practice. No ethical approval was required as it does not involve prospective interventional research or systematic collection of identifiable patient data. Where clinical images or patient information are included, informed consent for publication of anonymised clinical data and photographs was obtained from the patients. The authors confirm that the ethical policies of the journal, as stated in the author guidelines, have been followed.

## Consent

Written informed consent was obtained from all patients for participation in the treatment procedures and for the use of their clinical data for publication purposes, and the publication of their clinical photographs. Patients approved the use of their images for scientific purposes, and all reasonable efforts have been made to protect patient anonymity.

## Conflicts of Interest

All authors received honoraria from Galderma in relation to their participation in the expert consensus development. The funding source had no role in the study design, data interpretation, or manuscript preparation. Wei Thian Poh is an employee of Galderma.

## Supporting information


**Appendix A.** Summary of evidence extracted from eligible studies for advisory statement development.
Results for advisory statements and panel feedback.

## Data Availability

The data that supports the findings of this study are available in the Appendix A of this article.
