# Supplementary material for: Clinical Experience of Poly‐L‐Lactic Acid (Sculptra) Across Different Patient Profiles Using the AART Methodology and Expert Consensus on Minimizing the Risk of Complications—The Malaysian Perspective
Source: J Cosmet Dermatol. 2026 Sep 9;25(9):e71165. doi: 10.1111/jocd.71165 (PMC13558784; doi:10.1111/jocd.71165)
Supplement: Supplementary file 1 [file JOCD-25-e71165-s001.docx]

**Supplementary Appendix A**

**Summary of Evidence Extracted from Eligible Studies for Advisory Statement Development**

| **Statement** | **Evidence extracted from selected articles** |
| --- | --- |
| **1. Risk Zones** |  |
| The anatomical regions, risk levels, injection planes, and techniques outlined in the table & figure below are recommended for the safe use of biostimulators. Following these guidelines can help minimise the risk of adverse events. | - Risk classification is generally determined by the distribution of facial arteries and their anastomoses. High-risk zones include the frontal, periorbital, perioral and nasal regions. The temporal region, medial cheek, nasolabial folds and the mandibular area are considered intermediate risk. The lateral and middle cheek is classified as a low-risk zone (1,2). |
|  | - The temporal region, cheeks, and mandibular area are common sites for biostimulator use (3,4). - In contrast, biostimulator injections are not recommended in the frontal, periorbital, nasal, lip, and perioral regions (3-6). |
|  | - Several injection techniques are advised based on the appropriate injection plane to enhance safety and efficacy (3,7). - The fanning technique is suggested for deep subcutaneous injections, such as in the cheeks and preauricular areas. For regions such as the medial maxillary area, zygomatic area, temples, prejowl sulcus, and chin, the fanning, depot, or retrograde linear threading techniques are recommended (7). |
| **2. Management of Nodules and Granuloma** |  |
| **2.1 Recognizing the Symptoms**: Nodules can be visible or palpable but not visible, characterized by their firm, localized nature and often resulting from technical errors or uneven product distribution. They must be carefully differentiated from granulomas, which are clinically visible as subcutaneous nodules and result from an excessive inflammatory response. Nodules usually occur within the first 3 months after injection, while granulomas typically appear later. | - A nodule can be visible or nonvisible and may arise as a result of technical error, uneven distribution of the poly-L-lactic acid in the suspension, or uneven dispersal in the injected areas. It is distinguished by its indurated nature and separation from the surrounding soft tissue. It tends to remain the same size until it is absorbed, treated, or removed (5). |
|  | - Nodules and granulomas are significant complications that require differentiation. Nodules generally result from the accumulation of the product, manifesting as palpable lumps beneath the skin. Conversely, granulomas arise due to an excessive inflammatory response to the product material, marked by a more intense immune reaction (3, 8). |
| **2.2 Diagnosis of Nodules and Granuloma:** A distinction should be made based on the onset of nodules after injection, whether they are inflammatory or non-inflammatory, and the type of biostimulator used should be identified. Accurate diagnosis through detailed clinical history taking and examination is essential for appropriate management. | **Classification of nodules:**   - Nodules can be classified according to the time of appearance following injection as either early-onset (within 1 day to 3 months) or late-onset (> 3 months) (5,12). - Nodules may be inflammatory or non-inflammatory in nature (13). Additionally, the type of biostimulator used should be identified, as it may influence the underlying pathophysiology and management (14). |
|  | - **Early onset nodules and late onset nodules:** Early non- erythematous and nonpainful nodules appearing soon after administering an injection is likely the result of an uneven distribution of a product that has been injected too superficially or in too large a quantity (15). These nodules should be distinguished from inflammatory processes that may be due to an infection or immunological reaction (16,17). - The nodules tend to be noninflammatory, painless, confined to the site of injection and static in size. Early inflammatory nodules may present a bacterial infection and this should be excluded with microbiological testing (18). - The most probable diagnosis of late-onset nodules is granuloma. Before starting the treatment of any late-onset nodule, the diagnosis should be confirmed and the product known (14). - Early-onset nodule (onset 1-3 months after injection) responds poorly to corticosteroids, and a late-onset nodule (appearing 6-36 months after injection) responds well to intralesional corticosteroid injection (12). |
| **2.3 Management of Nodules and Granuloma:** Management of nodules depends on their onset and clinical characteristics. Complete resolution of early-onset nodules is achievable with appropriate treatment. Treatment recommendations for nodules and granulomas are shown in the table below. | **Wait and see:**   - Wait and see attitude is recommended, especially if nodule is nonvisible. Spontaneous resolution can occur (4). - Physicians should watch and wait or treat with oral corticosteroids if the nodules are visible. Meanwhile, patients can be reassured if the nodules are non-visible (19). |
|  | **Adequate massage:**   - A mass presenting at the time or within hours of injection is often due to product misplacement or edema (20). Massage by mechanical displacement and diffusion will help to reduce and disperse the product (21). - If the patient feels uncomfortable with the nodules, vigorous massage, with or without mechanical disruption (eg: subcision) may help (4). |
|  | **Normal saline injection:**   - Early nodules that appear within several weeks of treatment typically represent clustering of products. The coalescence of these microparticles can be disrupted using several techniques, including needle fragmentation of the nodule followed by injection of 0.9% saline to hydrate and redistribute the particles, combined with aggressive massage (5). - Study reported successful management using two injections of 1.0 mL of 0.9% saline solution at one-week intervals, along with local massage (22). - For non-inflammatory nodules, intralesional normal saline injection with lidocaine may be used (19, 23). |
|  | **Corticosteroid:**   - When the nodules appear after a few hours or days, they should be considered as an infection or immunological reaction and treated accordingly (24). Treatment of choice is systemic corticosteroids, but only after excluding an infectious cause (25). - Late nodules that appear several months after injection may be treated with an intralesional steroid (triamcinolone, 10 mg/ cc) and massage (5). |
|  | **Granuloma:**   - Different treatment approaches have been proposed for the treatment of granuloma. Corticosteroids or 5FU (5- fluorouracil) injections are very useful in many cases by diminishing the inflammatory response (17). - Steroid therapy (i.e., intralesional, intramuscular, and oral) with or without immune-modulating medications is the mainstay of intervention (5). The intralesional injection of triamcinolone is the treatment of choice for the late-onset PLLA nodules (26,27) and inflammatory granulomas (23). - Intralesionally injected 5-fluorouracil, alone or in combination with triamcinolone acetonide or betamethasone, are among other approaches demonstrated to be highly effective (28). - In addition, intense pulsed light can be a useful adjunct for the treatment of engorged capillaries (5). - Failure to respond to these treatments may mandate local excision of the lesion (26,27). Surgical removal should only be considered when non-surgical methods do not result in satisfactory outcomes, with an emphasis on preserving as much normal tissue as possible to minimise both functional and aesthetic complications (29). |
| **3. Preventative Measure of Nodules and Granuloma** |  |
| **3.1 Facial Anatomy:** A thorough understanding of facial anatomy is essential to reduce the risk of adverse events associated with biostimulator injections. | - Understanding facial anatomy is crucial to avoid injecting into or too close to blood vessels (3,30). - Injection sites associated with the most favorable outcomes are dynamically stable, with sufficient dermal thickness to allow a proper depth of injection (3). |
| **3.2 Injection Techniques:** Injection into the subcutaneous or deep dermis layer (depending on which type of biostimulator) is recommended to reduce the risk of nodule formation. Intramuscular and intradermal injections should be avoided. Improved injection techniques help minimise the risk of adverse events. | - Intramuscular and intradermal injections should be avoided (7). - Product placement in the subcutaneous or periosteal plane can help reduce the incidence of nodule formation (30). - Papule and nodule formation decreased with better experience of PLLA and highly evolved injection technique; the majority disappearing spontaneously within a year (4). |
| **4. Preventative Measure for Vascular Occlusion** |  |
| To avoid vascular occlusion, physicians must have a solid understanding of facial vascular anatomy and safe injection techniques. High-risk areas such as the glabella, nose, nasolabial fold, and forehead should be approached with caution or avoided if necessary. Using small injection volumes, blunt cannulas larger than 27G, slow injection techniques, and aspirating before injection can help reduce risk (depending on the type of biostimulator used). It is also important to review the patient’s history, as previous procedures may have altered arterial anatomy. | - Knowledge of both location and depth of facial vasculature is critical for avoiding vascular occlusion with injectable (35,44). - Physician must work to minimise risks by adopting a safer injection technique and having a thorough knowledge of three-dimensional facial anatomy. They must be able to recognise the clinical presentation of ischaemia, having clear protocols and referral pathways in place (2). - High-risk areas for vascular occlusion include the glabella and nasolabial folds. However, cases of tissue necrosis involving the lips and nose have also been reported (11,50,51). - The glabella, nose, nasolabial fold, and forehead are usually considered as the high-risk areas for blindness complications (52,53). - Areas located near the facial artery and angular artery, particularly along the nasolabial fold, nose, and glabella, are considered high risk. In these regions, product placement should be performed either deeply in the supra-periosteal plane using a needle or more superficially using a blunt cannula (39). - Risk factors for vascular occlusion include large volume bolus injection, use of small sharp needles, deeper injection planes (e.g. subcutaneous level) and high-pressure injections (43). - Small sharp needles are more likely to penetrate vascular lumens when compared to larger bore needles or blunt cannulas. The use of blunt cannulas may help reduce the risk of vascular injury (39). - Previous scarring can stabilize and immobilize arteries, making them more prone to penetration by needles (39). One study reported a case of vascular complication following PLLA injection in a patient with a history of rhinoplasty. The authors suspected that the previous procedure may have compromised vascular anastomoses, thereby increasing the patient's susceptibility to ischaemia (33). - Preventative strategies include using small injection volumes, blunt cannulas larger than 27G, and slow injection techniques. Although aspiration prior to injection does not guarantee vascular safety, it is still recommended as a precautionary measure (39). |

**Results for Advisory Statements and Panel Feedback**

| **Statement** | **Results** | **Panel Feedback** |
| --- | --- | --- |
| **1. Risk Zones** |  | - Chin can be low risk - I do agree with the risk zone suggested. Perhaps on the temple region it could also be a green/yellow zone as this area is frequently treated with biostimulator. With a retrograde fanning order I don’t think it is that risky compared to the other areas. - I personally think the temporal fossa and lateral cheek are rather low risk areas if treatment are performed subcutaneously. - Suggest to go interfascial space to cover more layers in the temple - NLF Area or NLF Folds, i think this is risky zone due to we are doing subcuteanous injection - Marionette as well - usually we avoid putting sculptra in these areas, so need special mentioning of these zone? - Risky zone (need specify risk-? vascular risk zone or ?nodule risk zone) |
| **2. Management of Nodules and Granuloma** |  |  |
| **2.1 Recognizing the Symptoms**: |  |  |
| **2.2 Diagnosis of Nodules and Granuloma** |  | - Early onset of nodule with sign of inflammation is strongly recommended to start antibiotic without bacterial analysis - Strongly agree- this allows the practitioner to have a clearer idea and the mode of treatment. - For suspect infection it is often not practical to do bacteriology sampling. Empirical antibiotics is more practical - Excisional biopsy is the last step to confirm and treat granuloma. Mostly we don’t need histopathological confirmation to treat granuloma with intralesional steroid. - In my opinion I think what most practical that the physician can do is to try to disperse by using normal saline and evaluate first -rather than to jump straight to histopathological confirmation depending on the duration it developed. - biopsy to rule out granuloma is not always practical. Sign and symptom eg clnical history eg onset time can be acceptable |
| **2.3 Management of Nodules and Granuloma** |  | - Energy based device - recommend laser assisted lipolysis - Strongly agree-it needs to be followed by the sequence and surgical intervention will only come in as a last option if the above algorithm of treatment is not effective . |
| **3. Preventative Measure of Nodules and Granuloma** |  |  |
| **3.1 Facial Anatomy** |  | - Rarely patient may have ptosis of brow transiently when injecting PLLA with lignocaine over temporal region. - Strongly agree-knowing anatomy and skin condition is crucial in this matter as biostimulator reacts differently comparatively to for ex:ha fillers |
| **3.2 Injection Techniques:** |  | - Do not inject PLLA when patient was known to have nodule formation due to other biostimulator before. The chances to develop nodule is high. - Strongly agree- precision,technique and understanding the outcome is important to get the desired outcome - terms subcutenous, subdermal, deep dermis, hypodermis-sometimes means different things to different physician, Its better we have a diagram to show what we mean - Cannula can minimise the risk for this concern |
| **4. Preventative Measure for Vascular Occlusion** |  |  |

Advisory Panel Agreement on Risk Zones (Total Response = 6)

|  |  |
| --- | --- |
| Strongly Disagree | 0 |
| Disagree | 0 |
| Neutral | 0 |
| Agree | 3 |
| Strongly Agree | 3 |

Advisory Panel Agreement on Recognizing The Symptoms Of Nodules And Granuloma (Total Response = 6)

|  |  |
| --- | --- |
| Strongly Disagree | 0 |
| Disagree | 0 |
| Neutral | 1 |
| Agree | 1 |
| Strongly Agree | 4 |

Advisory Panel Agreement on Diagnosis of Nodules and Granuloma (Total Response = 6)

|  |  |
| --- | --- |
| Strongly Disagree | 0 |
| Disagree | 0 |
| Neutral | 0 |
| Agree | 2 |
| Strongly Agree | 4 |

Advisory Panel Agreement on Management Of Nodules And Granuloma (Total Response = 6)

|  |  |
| --- | --- |
| Strongly Disagree | 0 |
| Disagree | 0 |
| Neutral | 0 |
| Agree | 0 |
| Strongly Agree | 6 |

Advisory Panel Agreement on Preventative Measure Of Nodules And Granuloma: Facial Anatomy (Total Response = 6)

|  |  |
| --- | --- |
| Strongly Disagree | 0 |
| Disagree | 0 |
| Neutral | 0 |
| Agree | 0 |
| Strongly Agree | 6 |

Advisory Panel Agreement on Preventative Measure Of Nodules And Granuloma: Injection Techniques (Total Response = 6)

|  |  |
| --- | --- |
| Strongly Disagree | 0 |
| Disagree | 0 |
| Neutral | 0 |
| Agree | 1 |
| Strongly Agree | 5 |

Advisory Panel Agreement on Preventative Measure For Vascular Occlusion (Total Response = 6)

|  |  |
| --- | --- |
| Strongly Disagree | 0 |
| Disagree | 0 |
| Neutral | 0 |
| Agree | 1 |
| Strongly Agree | 5 |

**References**

1. van Loghem, J., Funt, D., Pavicic, T., Goldie, K., Yutskovskaya, Y., Fabi, S., ... & Saeed, P. (2020). Managing intravascular complications following treatment with calcium hydroxylapatite: an expert consensus. Journal of cosmetic dermatology, 19(11), 2845-2858.
2. Murray, G., Convery, C., Walker, L., & Davies, E. (2021). Guideline for the management of hyaluronic acid filler-induced vascular occlusion. The Journal of Clinical and Aesthetic Dermatology, 14(5), E61.
3. Vleggaar D, Fitzgerald R, Lorenc ZP. Understanding, avoiding, and treating potential adverse events following the use of injectable poly-L-lactic acid for facial and nonfacial volumization. J Drugs Dermatol 2014;13(4 Suppl):s35-9.
4. Alessio, R., Rzany, B., Eve, L., Grangier, Y., Herranz, P., Olivier-Masveyraud, F., & Vleggaar, D. (2014). European expert recommendations on the use of injectable poly-L-lactic acid for facial rejuvenation. Journal of drugs in dermatology: JDD, 13(9), 1057-1066.
5. Lam, S. M., Azizzadeh, B., & Graivier, M. (2006). Injectable poly-L-lactic acid (Sculptra): technical considerations in soft-tissue contouring. Plastic and reconstructive surgery, 118(3S), 55S-63S.
6. Butterwick, K., & Lowe, N. J. (2009). Injectable poly-L-lactic acid for cosmetic enhancement: learning from the European experience. Journal of the American Academy of Dermatology, 61(2), 281-293.
7. Bartus, C., Hanke, W. C., & Daro-Kaftan, E. (2013). A decade of experience with injectable poly-L-lactic acid: a focus on safety. Dermatologic Surgery, 39(5), 698-705.
8. Lee, J. M., & Kim, Y. J. (2015). Foreign body granulomas after the use of dermal fillers: Pathophysiology, clinical appearance, histologic features, and treatment. Archives of Plastic Surgery, 42, 232–239.
9. Cassuto, D., & Sundaram, H. (2013). A problem-oriented approach to nodular complications from hyaluronic acid and calcium hydroxylapatite fillers: Classification and recommendations for treatment. Plastic and Reconstructive Surgery, 132(4 Suppl. 2), 48S–58S.
10. Christensen, L. H. (2009). Host tissue interaction, fate, and risks of degradable and nondegradable gel fillers. Dermatologic Surgery, 35(Suppl. 2), 1612–1619.
11. DeLorenzi, C. (2014). Complications of injectable fillers, Part 2: vascular complications. Aesthetic Surgery Journal, 34(4), 584–600.
12. Jin, Y. T., Chang, J. Y. F., Lang, M. J., & Chiang, C. P. (2022). Cosmetic materials-induced foreign body granuloma at the lower lip. Journal of Dental Sciences, 17(1), 586-588.
13. Redaelli A, Forte R (2009) Cosmetic use of polylactic acid: report of 568 patients. J Cosmet Dermatol 8:239–248)
14. Modarressi, A., Nizet, C., & Lombardi, T. (2020). Granulomas and nongranulomatous nodules after filler injection: different complications require different treatments. Journal of Plastic, Reconstructive & Aesthetic Surgery, 73(11), 2010-2015.
15. Lemperle, G., Gauthier-Hazan, N., Wolters, M., Eisemann-Klein, M., Zimmermann, U., & Duffy, D. M. (2009). Foreign body granulomas after all injectable dermal fillers: Part 1. Possible causes. Plastic and Reconstructive Surgery, 123(6), 1842–1863.
16. Narins, R. S., Coleman, W. P., & Glogau, R. G. (2009). Recommendations and treatment options for nodules and other filler complications. Dermatologic Surgery, 35(Suppl. 2), 1667–1671.
17. Alam, M., & Dover, J. S. (2007). Management of complications and sequelae with temporary injectable fillers. Plastic and Reconstructive Surgery, 120(6 Suppl.), 98S–105S.
18. Chiang, Y. Z., Pierone, G., & Al-Niaimi, F. (2016). Dermal fillers: pathophysiology, prevention and treatment of complications. Journal of the European Academy of Dermatology and Venereology, 31(3), 405–413.
19. Avelar, L., Ong, A., Ong, D., Wai, A. C. S., Wai, A. Y. T., Sungkyu, J., ... & Prygova, I. (2023). Consensus recommendations on the use of injectable poly‐l‐lactic acid in Asian patients. Journal of Cosmetic Dermatology, 22(12), 3223-3231.
20. Rzany, B., & DeLorenzi, C. (2015). Understanding, avoiding, and managing severe filler complications. Plastic and Reconstructive Surgery, 136(5), 196S–203S.
21. Funt, D., & Pavicic, T. (2015). Dermal fillers in aesthetics: An overview of adverse events and treatment approaches. Plastic Surgical Nursing, 35(1), 13–32.
22. Bravo, B. S. F., & Carvalho, R. M. (2021). Safety in immediate reconstitution of poly-L-lactic acid for facial biostimulation treatment. Journal of Cosmetic Dermatology, 20(5), 1435–1438.
23. Nikolis, A., Avelar, L. E., & Enright, K. M. (2021). Evaluation of cannula safety in injection of poly-L-lactic acid. Clinical, Cosmetic and Investigational Dermatology, 14, 615–622.
24. King, M., Bassett, S., Davies, E., & King, S. (2016). Management of delayed onset nodules. Journal of Clinical and Aesthetic Dermatology, 9(11), E1–E5.
25. Homsy, A., Rüegg, E. M., Jandus, P., Pittet-Cuénod, B., & Modarressi, A. (2017). Immunological reaction after facial hyaluronic acid injection. Case Reports in Plastic Surgery and Hand Surgery, 4(1), 68–72.
26. Hamilton, D. G., Gauthier, N., & Robertson, B. F. (2008). Late-onset, recurrent facial nodules associated with injection of poly-L-lactic acid. Dermatologic Surgery, 34, 123e6.
27. Lowe, N. J., Maxwell, C. A., & Patnaik, R. (2005). Adverse reactions to dermal fillers: Review. Dermatologic Surgery, 31, 1616e25.
28. Lemperle, G., & Gauthier-Hazan, N. (2009). Foreign body granulomas after all injectable dermal fillers: Part 2. Treatment options. Plastic and Reconstructive Surgery, 123(6), 1864–1873.
29. Choi, M., Choi, W. Y., Kim, J. M., Cheon, J. S., & Yang, J. Y. (2024). Foreign-body granuloma formation in the lower eyelid after injection of poly-D, L-lactic acid as a collagen stimulator: a case report. Archives of Aesthetic Plastic Surgery, 30(4), 137-140.
30. Levy, L. L., & Emer, J. J. (2012). Complications of minimally invasive cosmetic procedures: prevention and management. Journal of cutaneous and aesthetic surgery, 5(2), 121-132.
31. Beleznay, K., Carruthers, J. D. A., Humphrey, S., & Jones, D. (2015). Avoiding and Treating Blindness From Fillers. Dermatologic Surgery, 41(10), 1097–1117.
32. Wu, C. W., & Wu, H. J. (2021). Retinal artery occlusion following cosmetic injection of poly-L-lactic acid. Taiwan Journal of Ophthalmology, 11, 317–320.
33. Roberts, S. A. I., & Arthurs, B. P. (2012). Severe visual loss and orbital infarction following periorbital aesthetic poly-(L)-lactic acid (PLLA) injection. Ophthalmic Plastic and Reconstructive Surgery, 28(3), e68–e70.
34. Kim, J. H., Ahn, D. K., Jeong, H. S., & Suh, I. S. (2014). Treatment algorithm of complications after filler injection: Based on wound healing process. Journal of Korean Medical Science, 29(Suppl. 3), S176–S182.
35. Loh, K. T., Chua, J. J., Lee, H. M., et al. (2016). Prevention and management of vision loss relating to facial filler injections. Singapore Medical Journal, 57(8), 438–443.
36. Lazzeri, D., Agostini, T., Figus, M., Nardi, M., et al. (2012). Blindness following cosmetic injections of the face. Plastic and Reconstructive Surgery, 129, 994–1012.
37. Carruthers, J. D., Fagien, S., Rohrich, R., Weinkle, S., et al. (2014). Blindness caused by cosmetic filler injection: A review of cause and therapy. Plastic and Reconstructive Surgery, 134, 1197–1201.
38. Urdiales-Gálvez, F., Delgado, N. E., Figueiredo, V., Lajo-Plaza, J. V., Mira, M., Moreno, A., … Rebenaque, C. V. (2018). Treatment of soft tissue filler complications: Expert consensus recommendations. Aesthetic Plastic Surgery, 42(2), 498–510.
39. Parada, M. B., Cazerta, C., Afonso, J. P. J. M., & Nascimento, D. I. S. D. (2016). Overview and management of filler complications. Surgical & Cosmetic Dermatology, 8(4), 342–351.
40. DeLorenzi, C. (2017). New high-dose pulsed hyaluronidase protocol for hyaluronic acid filler vascular events. Aesthetic Surgery Journal, 37(7), 814–825.
41. Sclafani, A. P., & Fagien, S. (2009). Treatment of injectable soft tissue filler complications. Dermatologic Surgery, 35, 1672–1680.
42. Beleznay, K., Humphrey, S., Carruthers, J., & Carruthers, A. (2014). Vascular compromise from soft tissue augmentation: Experience with 12 cases and recommendations for optimal outcomes. Journal of Clinical and Aesthetic Dermatology, 7, 45–51.
43. Cohen, J. L., Biesman, B. S., Dayan, S. H., DeLorenzi, C., Lambros, V. S., Nestor, M. S., et al. (2015). Treatment of hyaluronic acid filler-induced impending necrosis with hyaluronidase: Consensus recommendations. Aesthetic Surgery Journal, 35(7), 844–849.
44. Yuan, J. T., Chang, T. W., Yu, S. S., & Arron, S. T. (2017). Mental artery occlusion from poly-L-lactic acid injection at the lateral chin. Dermatologic Surgery, 43(11), 1402–1405.
45. Nettar, K., & Maas, C. (2012). Facial filler and neurotoxin complications. Facial Plastic Surgery, 28(3), 288–293.
46. Signorini, M., Liew, S., Sundaram, H., De Boulle, K. L., Goodman, G. J., Monheit, G., et al. (2016). Global aesthetics consensus group. Global aesthetics consensus: Avoidance and management of complications from hyaluronic acid fillers—evidence- and opinion-based review and consensus recommendations. Plastic and Reconstructive Surgery, 137(6), 961e–971e.
47. Schanz, S. (2002). Arterial embolization caused by injection of hyaluronic acid (Restylane). British Journal of Dermatology, 146, 928–929.
48. Beer, K., Downie, J., & Beer, J. (2012). A treatment protocol for vascular occlusion from particulate soft tissue augmentation. Journal of Clinical and Aesthetic Dermatology, 5(5), 44–47.
49. Quach, B., & Clevens, R. A. (2024). Complications of injectables. Atlas of the Oral and Maxillofacial Surgery Clinics of North America, 32(1), 57–63.
50. Kassir, R., Kolluru, A., & Kassir, M. (2011). Extensive necrosis after injection of hyaluronic acid filler: Case report and review of the literature. Journal of Cosmetic Dermatology, 10(3), 224–231.
51. Burt, B., Nakra, T., Issacs, D. K., & Goldberg, R. A. (2010). Alar necrosis after facial injection of hyaluronic acid. Plastic and Reconstructive Surgery, 125(5), 199–200.
52. Park, K. H., Kim, Y. K., Woo, S. J., Kang, S. W., Lee, W. K., Choi, K. S., et al. (2014). Iatrogenic occlusion of the ophthalmic artery after cosmetic facial filler injections: A national survey by the Korean Retina Society. JAMA Ophthalmology, 132, 714–723.
53. Lee, J. S., Kim, J. Y., Jung, C., & Woo, S. J. (2020). Iatrogenic ophthalmic artery occlusion and retinal artery occlusion. Progress in Retinal and Eye Research, 100848.
